# A case report of a primary lymphoma of the tongue presenting as trigeminal neuralgia

**DOI:** 10.1186/s40463-019-0360-9

**Published:** 2019-08-05

**Authors:** Andrew J. Arifin, Selay Lam, S. Danielle MacNeil

**Affiliations:** 1 Division of Radiation Oncology, Department of Oncology, London Regional Cancer Program, 800 Commissioners Road East, London, Ontario N6A 5W9 Canada; 2 Division of Hematology, Department of Medicine, London Regional Cancer Program, 800 Commissioners Road East, London, Ontario N6A 5W9 Canada; 3 Department of Otolaryngology-Head & Neck Surgery, London Health Sciences Centre, 800 Commissioners Road East, London, Ontario N6A 5W9 Canada

**Keywords:** Lymphoma, Oral cavity, Head and neck, Trigeminal nerve, Perineural spread

## Abstract

**Background:**

Primary lymphoma of the oral cavity is a rare phenomenon. Herein we describe a unique presentation of lymphoma of the tongue which initially manifested as trigeminal neuralgia.

**Case presentation:**

A 63-year-old female experienced a 10-month history of paresthesia and neuropathic pain involving the left tongue and mandibular area of her face. Investigations including imaging and biopsy revealed primary lymphoma of the tongue with extensive perineural spread. The patient underwent standard chemotherapy with complete radiological response, but minimal recovery of the affected neurological functions.

**Conclusion:**

This case highlights an unusual presentation of a rare disease leading to a delay in diagnosis and the importance of a complete workup for trigeminal neuropathy.

## Background

The most common malignancy in the oral cavity is squamous cell carcinoma, which typically develops as a red or white lesion of the oral mucosa. Lymphomas are the most common non-epithelial malignancy in the oral cavity, though considered a rare entity, representing 3–5% of all reported cases of lymphoma [1]. The most frequent reported symptom of oral cavity lymphomas is local painless swelling with or without ulceration, though they can present in various ways and mimic other diseases [2]. Herein we report an unusual manifestation of this uncommon malignancy, presenting as trigeminal neuralgia.

## Case presentation

A 63-year-old previously healthy Caucasian woman was evaluated for a 10-month history of paresthesia and neuropathic pain involving the left tongue and left mandibular area of her face. She was initially treated for presumed trigeminal neuralgia, and neuropathic pain agents helped her marginally. Due to a lack of response to treatment, a magnetic resonance (MR) scan with gadolinium contrast of her head was ordered by an otolaryngologist. The scan showed abnormal enhancement in the left Meckel cave along the course of the mandibular nerve with involvement through the foramen ovale, inferior temporal fossa, and medial pterygoid muscle. She was referred to a neuro-oncologist due to concerns that her neuropathy was related to metastases. Computed tomography (CT) scans with intravenous and oral contrast of the head/neck, thorax, and abdomen/pelvis initially did not show evidence of malignancy. During the course of the investigations, the patient was found to have a left-sided tongue mass on physical examination. She was referred to an otolaryngology-head and neck surgeon for work-up of her tongue lesion.

The patient did not recall the mass being present prior to her seeing the neuro-oncologist. She denied any pain associated with the mass. Review of systems, including constitutional symptoms, was otherwise negative. Examination of the head and neck demonstrated numbness of her left tongue and left mandibular area of her face. The patient did not report any changes to her sense of taste or hearing. Tongue and facial movement were preserved bilaterally. There was no facial droop. Intraoral examination did not reveal any visible masses or mucosal changes. Palpation of the tongue demonstrated a 1 × 2 cm mass deep to the mucosa that felt rubbery without overlying mucosal changes. The tonsils and uvula were normal. Lymphadenopathy of the head and neck were not appreciated on exam.

**Fig. 1:**
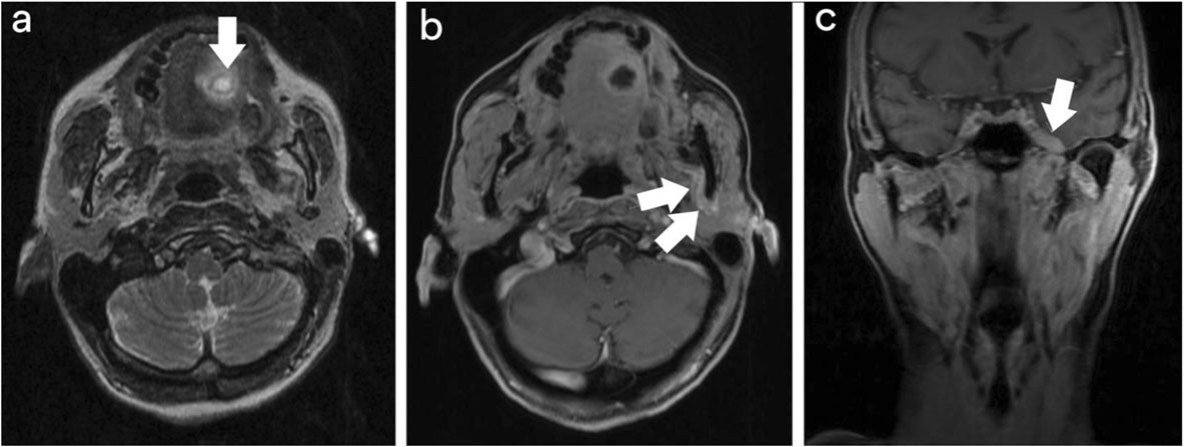
Representative MR neck images post-incisional biopsy. **a** T2-weighted axial MR sequence demonstrating the location of the primary lesion (arrow). **b** T1-weighted axial MR sequence post-gadolinium administration demonstrating asymmetric thickening posteromedial to the left ramus of mandible (arrows), corresponding to an area of anastomosis between cranial nerves V and VII. **c** T1-weighted coronal MR sequence post-gadolinium administration demonstrating asymmetric thickening along Meckel cave (arrow)

An incisional biopsy of the tongue mass was performed in clinic, which was read as diffuse large B cell-lymphoma (activated, post-germinal centre cell phenotype). A gadolinium contrast-enhanced MR scan of the neck was ordered to evaluate the lesion, which showed that the tongue mass exhibited perineural spread along the left lingual and inferior alveolar nerve, tracking along the V3 trigeminal branch to the left Meckel cave, in addition to perineural spread of the left facial nerve along the anastomosis with the auriculotemporal branch with the trigeminal nerve (Fig. 1). It was felt that the initial CT scan of the head and neck did not visualize the tongue lesion secondary to dental artifact. Based on the Ann Arbor staging classification, this patient was stage IIE. No lymph nodes were suspicious on CT or MR imaging, though a positron emission tomography (PET) scan revealed focal uptake in a left-sided level 2 lymph node measuring 5.8 mm with a maximum standardized uptake value of 8.2.

Incidentally, a mammogram (which was ordered as part of the initial whole-body investigation by the neuro-oncologist) and biopsy showed that she also had a synchronous invasive mammary carcinoma of the right breast.

**Fig. 2:**
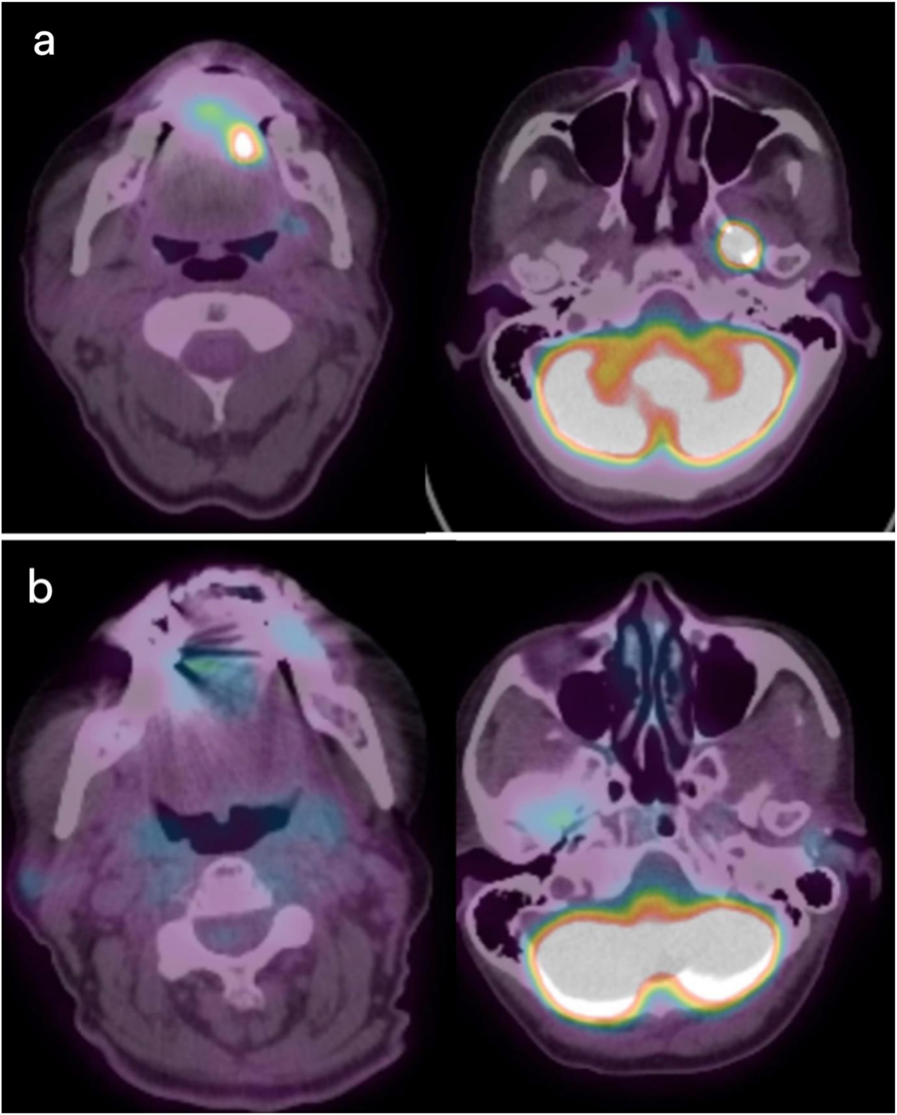
Representative fusion PET-CT images before and after R-CHOP chemotherapy. **a** Pre-treatment scans demonstrate intense uptake in the left oral tongue and left skull base. **b** Post-treatment scans demonstrate decreased uptake in previously intense areas

She was referred to hematology and general surgery for management of both primaries. After a multidisciplinary discussion, it was decided that she would undergo R-CHOP (rituximab, cyclophosphamide, hydroxydaunorubicin, oncovin, prednisone) chemotherapy for her lymphoma prior to definitive management of her breast cancer. She has currently completed 4 cycles of chemotherapy and positron emission tomography demonstrates complete disease response (Fig. 2). Following completion of her chemotherapy, she is planned to undergo breast surgery followed by adjuvant therapy. At the time of this report, the patient states that she has had minimal return of sensation to the left tongue and mandibular area to her face; however, she has had complete resolution of the left-sided facial pain with which she initially presented.

## Discussion

Lymphomas are the fifth most common cancer in Canada [3], and are classically divided into Hodgkin and Non-Hodgkin subtypes. Non-Hodgkin lymphoma (NHL) is a heterogenous group of neoplasms with a higher tendency of extranodal presentation compared to their Hodgkin counterpart. The most common subtype of NHL in the head and neck is diffuse large B-cell lymphoma [1]. As previously mentioned, the most frequent reported symptom of oral cavity lymphomas is local painless swelling with or without ulceration [2]. This case report is unique in highlighting an unusual presentation and location of this common malignancy.

The trigeminal nerve provides extensive sensory innervation to the face. The most well-known trigeminal nerve disorder is trigeminal neuralgia, which is classically defined as paroxysmal neuropathic pain along one or more of the trigeminal nerve distributions. The etiology in 80–90% of patients is a vascular anomaly which causes compression of the trigeminal nerve vasculature as it exits the pons. However, other conditions can produce similar symptoms, including malignancy, congenital malformations and multiple sclerosis. These non-classic conditions tend to cause neurologic symptoms in addition to the typical paroxysmal pain pattern [4]. In a joint guideline statement by the American Academy of Neurology and European Federation of Neurology Societies, the workup of trigeminal nerve dysfunction should include a complete history and physical examination, and may include head imaging and electrophysiologic testing [5]. In their review, a structural cause of trigeminal neuralgia was identified on imaging in 15% of cases. We performed a literature search on trigeminal neuropathy secondary to lymphoma and found a report of primary trigeminal nerve lymphoma [6], and a single case of sinonasal lymphoma with perineural spread [7].

Perineural invasion (PNI) is an important mechanism of malignant spread, in addition to direct invasion, hematologic and lymphatic spread. The mechanism of PNI is complex, involving a reciprocal relationship between host and tumour, in which a multitude of molecules in the nerve microenvironment facilitate metastasis [8]. PNI is commonly seen in head and neck squamous cell carcinoma, and salivary gland malignancies such as adenoid cystic carcinoma [9]. In contrast, PNI of lymphomas is not readily described in the literature.

## Conclusion

To our knowledge, this is the first case report of an oral cavity lymphoma presenting as trigeminal neuropathy. This case highlights the importance of a complete workup for trigeminal nerve disorder, as there is a broad differential for this condition. It also demonstrates an uncommon presentation for lymphoma, the fifth most common cancer in Canada.

## Data Availability

Not applicable.

## Abbreviations

CT: Computed tomography
MR: Magnetic resonance
NHL: Non-Hodgkin lymphoma
PET: Positron emission tomography
PNI: Perineural invasion
R-CHOP: Rituximab, cyclophosphamide, hydroxydaunorubicin, oncovin, prednisone
